# Research on gamified personality test design for wildlife conservation: integrating the theory of planned behavior and empathy

**DOI:** 10.3389/fpsyg.2026.1839884

**Published:** 2026-07-02

**Authors:** Bingni Li

**Affiliations:** Department of Visual Communication Design, Academy of Arts and Design, Tsinghua University, Beijing, China

**Keywords:** digital design, empathy, endangered species, personality test, theory of planned behavior

## Abstract

Currently, due to factors such as habitat loss and human activities, the populations of many endangered species have declined significantly, with some even facing the threat of extinction. Addressing this issue requires not only the involvement of experts but also the collective efforts of the public to support wildlife conservation. This study aims to explore the structural relationships between empathy towards endangered animals, attitudes towards conservation behavior, subjective norms, perceived behavioral control and behavioral intention, while constructing an extended framework of the Theory of Planned Behavior. Based on self-perception theory and empathy theory, participants were shown a demonstration of a personality test game designed by the authors prior to completing the questionnaire, followed by an online survey using a 5-point Likert scale. A total of 306 valid responses were ultimately collected. Structural equation modeling was employed to test the hypothesized relationships between variables. The model demonstrated good fit, confirming the reliability of the results. The findings indicate a strong public willingness to protect endangered animals, with 81% of respondents expressing a willingness to take action. The study found that empathy indirectly influences an individual’s behavioral intention through attitudes and perceived behavioral control (PBC). The findings of this study provide guidance for future endangered animal conservation efforts. For instance, government interventions should place greater emphasis on empathy-based strategies, including public awareness campaigns and educational programmes designed to encourage the public to consider the plight of endangered animals from the animals’ perspective. Furthermore, the government should provide accessible channels and resources. Such efforts can raise public awareness, thereby strengthening individuals’ behavioral intentions regarding wildlife conservation and ultimately promoting conservation behavior.

## 1 Introduction

Endangered species refer to wild animal species at risk of extinction due to internal biological factors and external influences such as human activities or natural disasters (IUCN Species Survival Commission, 2012). The protection of endangered species is crucial because all species are an indispensable part of the ecosystem. In the face of increasingly severe environmental challenges, the protection and management of wild animals has become a key link in maintaining the earth’s biodiversity and ecological balance (Gupta et al., 2023). However, the public’s awareness of this area is still insufficient. Many people believe that wildlife conservation has nothing to do with their daily lives or lack awareness of its importance. For example, the Malayan tapir, a well-known conservation species in Southeast Asia, has been reported to be poorly recognized among residents in Peninsular Malaysia, including a lack of knowledge regarding its ecological role and appropriate conservation measures (Jumaat et al., 2024).

Therefore, the core issue of the animal protection topic lies in the public’s psychological indifference. Psychological distance can be classified into four categories: (1) theoretical distance, (2) temporal distance, (3) spatial distance, (4) social distance. For individuals, to take action for the protection of endangered animals, such as participating in volunteer activities or making donations, there is a need to overcome this psychological distance (Santoso et al., 2025).

Earlier studies have investigated the role of social media in increasing public awareness of wildlife conservation (Wu et al., 2018). Their findings indicate that posts containing a higher portion of images in wildlife-related news tend to attract more engagement, indicating that visual content can significantly enhance the effectiveness of wildlife conservation communication.

The novelty of this study depends in using a test-game approach to evoke public empathy, thereby encouraging individuals to adopt a perspective-taking mindset. Although previous research has examined empathy in the context of wildlife conservation and has supported the association between attitudes towards animals and human empathy (Signal and Taylor, 2007), these studies mainly compared empathy levels between members of animal protection organizations and the general public. But they did not integrate the interactive game experience into the experimental environment. Therefore, existing studies have not fully explained the mechanism by which gamified design can stimulate public sympathy for wild animals.

Therefore, this study aims to explore how game-based interactive design affects the public’s behavioral intention for wildlife conservation and selects empathy as the initial variable in the framework of planned behavior theory (TPB). In order to achieve this goal, author selected a group of eight representative endangered animal species and designed a complete set of UI visuals for these endangered animals, which were included in the demonstration game as core visual and interactive stimulation materials. Endangered animals include armadels, spotted fish and Malay tadops. These species were selected because their protection status and their harms are closely related to human activities, such as hunting and habitat loss, so they are more representative and can also impress participants.

By embedding these animals within an interactive experience, this study seeks to examine how emotionally engaging digital environments can enhance awareness and promote pro-conservation intentions among the public. In line with the research objectives, this study proposes the following research questions:

*RQ1*: To what extent does game-based interactive design influence public attitudes towards wildlife conservation?

*RQ2*: To what extent does empathy towards endangered animals mediate the relationship between game experience and conservation attitudes?

*RQ3*: How do attitudes, subjective norms, and perceived behavioral control affect behavioral intentions towards wildlife conservation within the TPB framework?

## 2 Literature review and theoretical hypothesis

### 2.1 Personality quiz

As early as 1953, scholars applied personality research to the field of education (Cattell and Beloff, 1953). These early studies laid the methodological foundation for children’s personality assessment by introducing a factor-analytic approach to the development of standardized personality tests for children aged 10–16 (J.P.Q.). This line of work linked personality measurement with academic prediction and educational guidance, thereby establishing both theoretical and methodological foundations for the application of personality testing in educational contexts.

In recent years, international scholars have further explored the application of personality testing in student learning guidance. For example, Syahputra et al. (2024) developed a desktop application for Indonesian university students that integrates learning style assessments with personality tests. By enabling students to better understand their personal traits and align them with appropriate learning strategies, this tool has been shown to effectively support learning guidance and optimize teaching strategies. However, research on personality tests in the context of animal protection remains scarce.

Based on this, in the experiment, this study introduced a personality quiz game. Users’ responses were used to match corresponding endangered animals, and the results were presented in a story-like manner to enhance individuals’ emotional engagement and empathetic experience.

Therefore, personality quizzes can be conceptualized not only as assessment tools but also as identity-framing mechanisms that may facilitate psychological engagement with non-human entities.

### 2.2 Self-perception theory and self-projection

According to self-perception theory, proposed by Bern (1972), individuals infer their internal attitudes by observing their own behaviors and contextual cues. Similarly, individuals may rely on external cues and situational information to interpret their internal states. In the context of quiz-based interactions, assigned identities function as such cues, encouraging users to interpret the outcomes as self-relevant. This process provides a foundation for subsequent self-projection and empathic engagement. Therefore, in this study, the authors assigned a “creature that lived in a dangerous environment in the past” role to the participants. This method served as a form of identity cue, prompting them to interpret themselves based on this role. Drawing on perspective-taking theory (Underwood and Moore, 1982), adopting the viewpoint of another entity can reduce self–other distinction and facilitate both cognitive and affective empathy. In this context, participants are encouraged to simulate the experiences and vulnerabilities of the assigned creature, which in turn fosters empathic concern.

Self-projection refers to the ability to mentally simulate alternative perspectives, including those of other individuals or imagined identities (Buckner and Carroll, 2007). This enables users to imagine themselves in the position of a designated animal, thereby providing a cognitive basis for perspective-taking. This process is closely related to perspective-taking, which has been widely associated with the development of empathy.

In Cohen (2001) study, it was proposed that when we resonate with others, in fact, we are setting a role for ourselves based on the characteristics of the other person. Its meaning is that when we “act out” this role internally and become fully immersed in this imagined performance. This process explains why immersive role-playing in games can trigger genuine empathy and psychological transformation.

In previous studies related to animal protection, such as Ahn et al. (2016), where users were made to “become animals” in a virtual environment, it significantly enhanced their empathy for nature and the sense of integration between themselves and nature.

Furthermore, in the study by Tran et al. (2026), the team employed the method of designing probes and discovered through two prototypes—voice AI koalas and XR virtual koalas—that giving animals voices, images, and identities can effectively enhance people’s empathy and concern for non-human subjects. This study also pointed out that perspective transformation, anthropomorphic expression, and embodied presentation are key design ideas for stimulating empathy with animals and nature. Thus, it can be concluded that as long as an identity connection is established between humans and animals, it is sufficient to trigger genuine emotional engagement. Tsumura and Yamada (2023) research indicates that the emotional expression and perspective substitution of anthropomorphic characters can effectively enhance players’ empathy and concern for non-human subjects.

With the popularity of virtual environments (such as online games and social media), users can freely change their self-representation through digital avatars. Yee and Bailenson proposed the Proteus Effect, the name of which is derived from the Greek myth of the transformable god Proteus, comparing the flexibility of users achieving behavioral changes through avatars to the individual’s behavior unconsciously adapting to the external features of the virtual avatar (Yee and Bailenson, 2007). This study revealed that virtual characters in games can change players’ psychological states and behaviors, and this is unconscious, automatic, and takes effect immediately. In other words, “becoming who you are in the game” makes you unconsciously become that person. And this change is not influenced by others’ opinions. This proves that the game itself can change people’s psychological states, providing clear evidence for the virtual self-presentations in games to change users’ internal psychological states.

In this study, users will also experience a personality test game based on animal protection. In the game, users will be required to input personal information such as zodiac sign, gender, and birthplace. Although this task seems random, it is a simple way for participants to obtain a specific role positioning. Once the role is assigned, people tend to interpret situations from the perspective of that role, which helps cultivate the ability of empathy and enhance emotional engagement (Cohen, 2001).

Previous research suggests that empathy can evoke altruistic motivation, that is, the willingness to engage in helping behaviors. The empathy–altruism hypothesis proposes that empathic concern for others can generate a motivation to help, independent of self-interest (Batson et al., 1991). Taken together, these findings indicate that personality-test-based games may have a direct effect on eliciting empathy. Therefore, this study adopts a personality-test game as a tool to stimulate empathy among the public.

This study is grounded in the assumption that identity-based gamification may facilitate empathy-related processes. Specifically, assigning a “past-life animal” introduces a form of identity framing, which may enhance user identification with the assigned species and encourage perspective-taking. Prior research in media psychology suggests that identification with non-human or mediated entities can support emotional engagement and empathic responses (Cohen, 2001).

Therefore, the game design in this study is theoretically expected to elicit empathy towards endangered animals through identification and perspective-taking mechanisms.

### 2.3 Animal protection intention

According to Ajzen (1991), a person’s intention is a mental state that denotes readiness to engage in a particular behavior. One’s actions are shaped by intentions, which are affected by attitudes, subjective norms, and perceived behavioral control.

In the context of animal protection intention, whether it is to enhance the wellbeing of animals for the benefit of humanity, or to protect animals for their own sake, many countries around the world have formulated policies to safeguard animals (Holst and Martens, 2016).

This study does not directly investigate the actual behaviors of public animal protection. Instead, it takes the willingness to protect animals as the outcome variable. This is because the measurability and measurability of animal protection willingness (Ajzen, 1985), as well as its predictive effect on future actual behaviors (Glasman and Albarracín, 2006), ensure that this study has more practical significance for society and the environment.

### 2.4 Empathy

Prior studies have verified that empathy serves not only as a critical element in human-to-human interactions but also plays a central role in shaping the connection between humans and other species.

Prato-Previde et al. (2022) Empathy refers to an individual’s ability to cognitively and emotionally relate to others, involving the understanding and internalization of another’s thoughts, emotions, and intentions. Through this process of perspective-taking, individuals are able to think from the standpoint of others, share their emotional experiences, and make more objective and appropriate evaluations of their situations, thereby providing emotional and behavioral support. Empathy has been widely recognized as a key psychological driver underlying altruistic behavior (Batson et al., 1981). The concept of empathy can be traced back to 19th-century German aesthetics. The German philosopher Theodor Lipps proposed that human cognition involves both the “self” and the “other-self,” and that understanding others requires empathy, a process also described as “self-objectification” (Chismar, 2020). This highlights empathy not only as a psychological phenomenon but also as a process of projecting one’s thoughts and feelings onto others. In addition, George Herbert Mead (1934) suggested that individuals can understand others by “taking the role of the other,” emphasizing the importance of role-taking in social cognition.

Taken together, empathy can be regarded as a human-centered design perspective and methodological approach, focusing on an individual’s ability to understand others’ experiences and adopt their perspectives. In recent years, empathy has also been employed as a key variable in studies on consumer behavior, such as research on the purchase of high animal-welfare beef (Washio et al., 2019), further supporting its applicability in related research contexts. In the study by Vu et al. (2026), which investigated Parasport Engagement Among Young Vietnamese Audiences, also based on extended TPB theory with empathy, which showd that empathy have an imapct on attitude, and indirectly affect behavior intention. Prior research has also demonstrated that empathic concern can lead to more positive attitudes towards others, even extending from an individual to a broader social group (Batson et al., 2002). As a result, it can be assumed that empathy will directly change public’s attitude.

In previous studies, Clowes and Masser (2012) demonstrated that emotional states (such as anxiety) significantly influence subjective norms. This proved that the SN is influenced by an individual’s emotional state.

Studies have shown that in the research conducted by Lee and Kweon (2013), when the sense of compassion is stronger, the degree of cooperation among groups will be higher. In other words, the sense of compassion will become stronger as the degree of cooperation within the group increases. Therefore, the sense of compassion can significantly predict the degree of cooperation within the group. As supported by De Leeuw et al. (2015), empathic concern significantly influences normative beliefs (both injunctive and descriptive), which in turn determine subjective norms (SN). This provides clear theoretical and empirical support for the path from empathy to SN. Furthermore, empirical evidence suggests that empathy can positively influence perceived behavioral control (Baciu et al., 2020). Within the Theory of Planned Behavior, PBC is determined by underlying control beliefs (Ajzen, 1991). This suggests that empathy may also exert an indirect effect on PBC by shaping individuals’ control-related cognitive appraisals.

Although previous studies have separately explored the roles of empathy, identity recognition, and perspective-taking in promoting prosocial and environmentally friendly behaviors, most of these studies were based on self-reporting survey designs and failed to fully reflect how these psychological processes are generated through interactions and identity-shaping experiences. In particular, there is currently a lack of relevant research to explore how the individual identity-setting mechanisms based on tests (such as interactive games based on personality tests) can serve as an intervention tool to prompt people to project themselves onto non-human entities (such as endangered animals) and develop empathy.

Furthermore, although the Theory of Planned Behavior has been widely applied in the fields of environmental and wildlife conservation, few studies have expanded this framework by positioning empathy as a systematic antecedent factor, thereby simultaneously influencing attitudes, subjective norms, and perceived behavioral control in interactive digital experiences.

Considering the factors outlined above, this study proposes the following hypotheses:

*Hypothesis 1 (H1)*: Empathy is positively associated with one individual’s attitude towards endangered animals’ protection.

*Hypothesis 2 (H2)*: Individuals with higher levels of empathy are more likely to be influenced by subjective norms.

*Hypothesis 3 (H3)*: Empathy positively influences perceived behavioral control.

### 2.5 The theory of planned behavior

**Figure 1:**
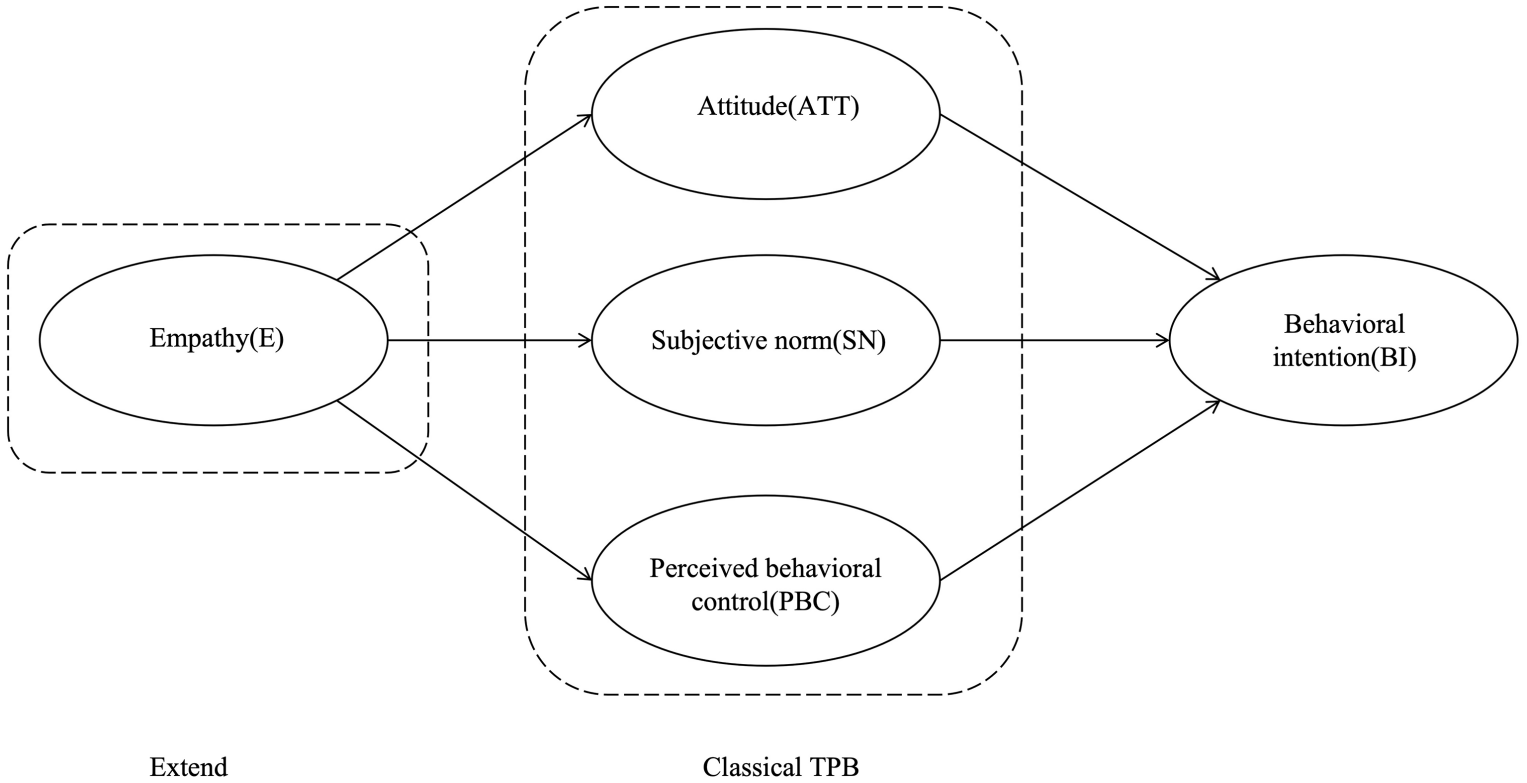
Based on the classical TPB extended framework.

In recent years, the Theory of Planned Behavior (TPB) has been widely utilized in studies related to wildlife conservation and environmental issues. For example, Zhang et al. (2022) employed the TPB model to examine public resistance to the consumption of wildlife, while (Lou et al., 2022) applied the model to examine individuals’ intentions towards waste sorting. In addition, more recent studies have extended the TPB framework to research on environmental protection, wildlife consumption, and animal welfare (Joo et al., 2025). These studies demonstrate that the TPB model is both scientifically grounded and applicable in the context of wildlife conservation. However, its application remains underexplored in the context of personality-test-based interventions and endangered species protection. This study therefore takes the TPB model as its theoretical foundation to investigate the effects of empathy on public attitudes and behavioral intentions. By extending the TPB framework, the presents study develops the following conceptual model (Figure 1).

The theory of planned behavior, extending TPB by incorporating empathy as an antecedent of attitude, SN and PBC, Behavioral intention is the cognitive representation of individuals’ willingness to participate in specific behavioral and is viewed as the forerunner of behavior (Ajzen, 1991). In the traditional Theory of Planned Behavior (TPB) framework, individuals’ behavioral intentions are mainly influenced by attitudes, subjective norms, and perceived behavioral control. However, in research contexts involving ethics, care, and inclusivity, emotional factors play an essential role.

In this research, I will use extended TPB model, treat empathy as an antecedent variable. Different from the traditional TPB model, the empathy variable (E) emotional resonance and perspective-taking ability, thereby generating genuine empathy, which will further act on the other core variables of the TPB model and affect the final behavioral intention.

The inclusion of empathy in the extended TPB framework is theoretically grounded in the Empathy–Altruism Hypothesis proposed by C. Daniel Batson (1991), which posits that empathic concern for others in need generates an altruistic motivational state directed towards relieving their suffering. This suggests that empathy is not merely an emotional response, but a motivational driver that can shape individuals’ evaluative judgments and action tendencies.

In the context of endangered species protection, empathy towards animals can therefore be understood as an upstream affective-motivational factor that influences attitudes and perceived behavioral control, thereby shaping behavioral intentions within the TPB framework.

Empathy is a key emotional driver that motivates individuals to develop caring intentions and engage in altruistic behaviors. Therefore, this study incorporates empathy as an antecedent variable to enhance the explanatory power of the theoretical framework in contexts involving care and prosocial behavior.

#### 2.5.1 Attitude

Attitude towards the behavior, defined as “the degree to which an individual holds a favorable or unfavourable evaluation of the behavior in question,” plays a critical role in influencing behavioral intention (Ajzen, 1985). The higher the individual’s positive evaluation of a behavior, the stronger their intention to perform that behavior will be; conversely, negative evaluation will significantly weaken the intention to carry out the behavior. In this study, the construct of “attitude” refers to the attitude towards the act of protecting animals, that is, whether the participants approve of the related behaviors of protecting animals.

On the one hand, if participants consider protecting animals to be a valuable, meaningful and worthwhile action to undertake, they are more likely to develop the willingness to actively participate in protection efforts. On the other hand, if they hold skeptical, indifferent or even negative attitudes towards the related actions, it will directly reduce their motivation to take protective actions. Therefore, attitude, as the core antecedent variable of the Theory of Planned Behavior, is regarded in this study as the key psychological basis for predicting an individual’s intention to engage in animal protection behaviors.

*Hypothesis 4 (H4)*: Attitude towards endangered animals’ protection positively correlates with one individual’s intention to engage in endangered animals’ protection behaviors.

#### 2.5.2 Subjective norm

Subjective norms refer to the support or opposition that an individual perceives from the social pressure regarding a certain behavior (Ajzen, 1985). The social pressure perceived by an individual often comes from “important others” (such as relatives, friends, classmates, or the public). In the majority of studies, subjective norms show the weakest effect on behavioral intentions (Yan, 2014).

This study aims to investigate whether subjective norms have an impact on people’s behavior of protecting animals. Here, subjective norms refer to the overall social atmosphere for protecting animals and the attitudes of each individual’s family and friends towards this act of protecting animals. When the majority of people around generally support and approve of animal protection actions, individuals are more likely to be positively motivated and thus develop the willingness to participate in related activities; conversely, this may reduce their inclination towards such actions.

*Hypothesis 5 (H5)*: Subjective norm positively correlates with an individual’s intention to involve in endangered animals’ protection behaviors.

#### 2.5.3 Perceived behavioral control

Perceived behavioral control is defined as an individual’s evaluation of their available resources and capabilities to perform a given behavior (Ajzen, 1985). In other words, refers to an individual’s perception of how easy or difficult it is to perform a particular behavior. While attitudes (ATT) and subjective norms (SN) reflect subjective factors, PBC also involves an evaluation of objective conditions (Yan, 2014). Protecting endangered animals depends on the public’s assessment of their own capabilities. It means whether individuals have the confidence that they can participate in animal protection activities. This confidence implies that individuals need to have the necessary conditions for protecting animals, which includes sufficient knowledge reserves about endangered animals, channels to engage in animal protection, time and money, etc. When individuals believe they have the necessary conditions or find it relatively easy to carry out animal protection actions, their willingness to participate and the likelihood of taking actual actions will significantly increase; conversely, if individuals perceive a lack of resources or numerous obstacles, it will directly restrict the occurrence of their protection actions.

*Hypothesis 6 (H6)*: Perceived behavioral control positively correlates with one individual’s intention to engage in endangered animals’ protection behaviors.

**Figure 2:**
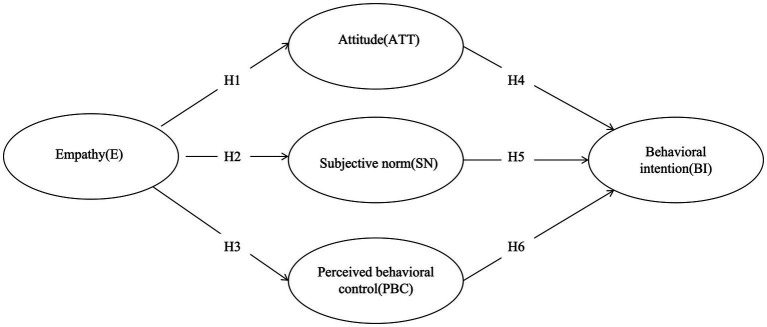
The research model.

The research model in this study was formulated based on the hypotheses outlined above (Figure 2).

## 3 Methodology

### 3.1 Endangered animal selection and classification

This study employed a personality-test-based game demo as the research material. A set of endangered animal species was selected as the core visual stimuli for the demo video. The selection aimed to ensure diversity across species categories (including mammals, birds, amphibians, and aquatic species), as well as recognizability and the potential to evoke empathy.

The selected species include the armadillo (Desbiez and Attias, 2022), saiga antelope (Hanski et al., 2023), Malayan tapir (Lim et al., 2025), California condor (Walters et al., 2010), blobfish (Petrescu-Mag, 2023) shoebill (Mirembe, 2018), quokka (Tores et al., 2007), and salamander (Luedtke et al., 2023). These animals were chosen based on their conservation status and their representativeness in raising public awareness of wildlife protection. Rather than representing specific taxa, they were intentionally selected to reflect a diverse range of species types, in order to enhance both recognizability and emotional engagement among participants.

The full list and details of the selected species are provided in Appendix A.

### 3.2 Participants

Data collection for this study was conducted from February 28 to March 9, during which a total of 316 participants were recruited. This study adopted a convenience sampling method to collect data. Data were collected from Chinese citizens using an online questionnaire distributed via Wenjuanxing.^1^ All questionnaire materials were presented in Chinese. Altogether, 316 participants voluntarily and anonymously completed the online questionnaire. Questionnaires completed in less than 40 s and those with identical responses across all items were excluded, 306were remained (143 females and 163males), with the effective rate of 96.8%.

### 3.3 Interaction design

Before beginning the questionnaire, participants were asked to watch a one-minute demo video demonstrating how to use the personality-test-based game. All participants viewed the same video, which did not include any voice-over narration.

**Figure 3:**
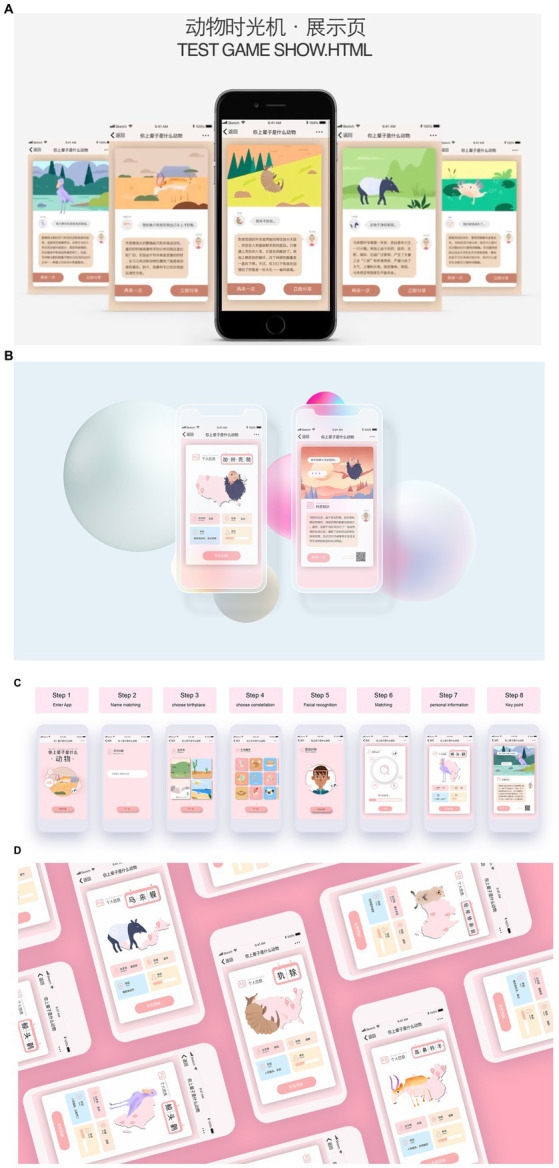
Personality quiz design. **(A)** Screenshot of demo video. **(B)** Information page. **(C)** Step introduction. **(D)** UI design.

The application, titled Animal Time Machine (Figure 3): Discover Your Past-Life Animal, guides users through a series of interactive steps. First, users open the app and are presented with an introductory interface, where they click “Start” to begin. They are then required to enter their name, select their place of birth and zodiac sign, and complete a facial recognition process. Based on this information, the application matches users with a corresponding endangered animal, such as the Malayan tapir or the blobfish. The result presents a randomly assigned “past-life animal.” The characteristics, habitat distribution and causes of harm of each species are introduced in detail on the last information page of the whole game.

Although the assignment may appear arbitrary, the use of a personalized identity label (“past-life animal”) is intended to create a minimal sense of personal relevance. Prior research suggests that even simple forms of personalization can increase identification and engagement, which may support perspective-taking in mediated contexts (Cohen, 2001).

Through this process, participants are exposed to information about endangered animals in a more engaging format than traditional educational materials. The use of a personality-test-based structure, combined with visual elements, presents the content in a more interactive way. In addition, assigning a “past-life animal” creates a personalized link between the user and a specific species. This form of personalization may encourage participants to consider the situation from the perspective of the animal, thereby supporting empathy towards endangered species.

### 3.4 Characteristic of the sample

The sample characteristics are shown in Table 1. The proportion of male and female respondents was similar. The majority of participants were below 40 years old, with the 20–29 age group representing 54.2% of the sample. In terms of the knowledge about endangered animals, only a small proportion of participants reported being very familiar with endangered animals (20.6%), while the majority demonstrated only limited or superficial knowledge. Regarding participation in wildlife protection activities, the majority participated only occasionally (70.6%), with few reporting frequent involvement (13.1%). Additionally, most participants had prior experience with quiz-based interactive games, with 74.2% indicating occasional use.

**Table 1:** Sample characteristics.

Measure	Category	Number	Percentage
Gender	Female	143	46.7%
	Male	163	53.3%
Education	Elementary school	1	0.3%
	Junior high school	14	4.6%
	Senior high school	65	21.2%
	College	200	65.4%
	Master/Ph.D.	26	8.5%
Age	Below 20	69	22.5%
	20–29	166	54.2%
	30–39	66	21.6%
	40–49	2	0.7%
	Above 50	3	1.0%
Knowledge about endangered animals			
	Very familiar	63	20.6%
	Slightly familiar	145	47.4%
	Have heard of but do not know	73	23.9%
	Almost do not know	25	8.2%
Frequency of participating in animal protection activities			
	Frequently	40	13.1%
	Occasionally	216	70.6%
	Never	50	16.3%
The frequency of using the test game			
	Frequently	42	13.7%
	Occasionally	227	74.2%
	Never	37	12.1%
Total		306	100%

### 3.5 Measurement instruments

The questionnaire is modified based on previous study (Washio et al., 2019), the questionnaire used in this study comprised two sections. The first section collected participants’ demographic information (e.g., gender, age, and education). The second section included five subscales with a total of 18 items, adapted from established and widely used measures to assess the five latent variables in this study (Ajzen, 2006). All subscales were measured using a five-point Likert scale, ranging from “strongly disagree” to “strongly agree.” The full version of the questionnaire can be found in Appendix A.

In the online survey, participants were first presented with a demonstration video of the game. After watching the video, they were explicitly instructed to proceed to the questionnaire (e.g., “After watching the video, please answer the following questions”), ensuring a clear temporal sequence between the intervention and the measurement.

Participants did not directly interact with the game but instead watched a short demonstration video, which may reduce the level of interactivity and immersion. However, this design ensured that all participants were exposed to a standardized stimulus, thereby minimizing variability in user experience across individuals.

The empathy-related subscale consisted of three items and was administered immediately after participants viewed the demonstration video. This scale was used as a manipulation check to assess whether the intervention elicited emotional responses towards endangered animals.

Prior to the formal survey, a pilot study involving 120 participants was carried out to assess the reliability and validity of the questionnaire. Based on the results, several items were identified as problematic and subsequently revised. The formal questionnaire was then finalized and distributed to participants, which helped ensure the accuracy and quality of the data.

### 3.6 Data analysis

Data analysis was carried out using SPSS 27.0 and AMOS 28.0. Participants were coded from 1 to 306. Descriptive statistical analyses were performed in SPSS, and structural equation modeling (SEM) was conducted in AMOS to evaluate model fit and examine the relationships among variables.

## 4 Results

The reliability and validity of the measurement model were assessed using standardized factor loadings, Cronbach’s alpha coefficients, convergent validity, discriminant validity, and model fit indices.

### 4.1 Instrument validation

According to Schumacker and Lomax (2004), Standardized factor loadings should generally exceed 0.50 for all items. All the items in this study meet this criterion well since their standardized factor loadings were all above 0.6 (ranging from 0.689 to 0.8), E4 and PBC4 were removed to improve the fitness of the construct (Segars, 1997).

First, Cronbach’s alpha was used to verify its reliability, when Cronbach’s alpha > 0.7, it can be proven to have high reliability (Cronbach, 1951). Cronbach’s alpha of five constructs were all above 0.7 (0.761–0.831) (Table 2). Table 2 shows that all coefficients meet the required thresholds, suggesting that the scale has good reliability.

**Table 2:** Results of construct validity and reliability analysis.

Latent variable	Measurement	Mean	Std. Dev	Factor loadings	*α*	CR	AVE
E	E1	4.04	0.89	0.691	0.764	0.766	0.522
	E2			0.746			
	E3			0.73			
ATT	ATT1	4.08	0.91	0.713	0.83	0.831	0.552
	ATT2			0.769			
	ATT3			0.772			
	ATT4			0.716			
SN	SN1	3.94	0.91	0.739	0.831	0.832	0.555
	SN2			0.674			
	SN3			0.8			
	SN4			0.761			
PBC	PBC1	3.74	0.95	0.775	0.761	0.765	0.521
	PBC2			0.693			
	PBC3			0.694			
BI	BI1	4.05	0.88	0.764	0.824	0.825	0.542
	BI2			0.689			
	BI3			0.769			
	BI4			0.72			

To assess the validity of the scale, the KMO coefficient was calculated and Bartlett’s sphericity test was performed. The KMO value was 0.912, and Bartlett’s test was significant (*p* < 0.001), implying that the data were suitable for factor analysis.

Convergent validity was evaluated using composite reliability (CR) and average variance extracted (AVE). CR values should exceed 0.70 and AVE values should be beyond 0.50 (Fornell and Larcker, 1981). In this study, all five constructs satisfied these two criteria (Table 2).

We evaluated discriminant validity through a comparison between the square root of the AVE and inter-construct correlations. A sufficient discriminant validity requires that the square root of each construct’s AVE be larger than its correlations with other constructs (Chin, 1998). The results indicate that the measurement model meets this criterion, demonstrating good discriminant validity (Table 3).

**Table 3:** Discriminate validity of the research model.

Construct	E	ATT	SN	PBC	BI
E	**0.722**				
ATT	0.489^**^	**0.742**			
SN	0.419^**^	0.464^**^	**0.744**		
PBC	0.484^**^	0.503^**^	0.486^**^	**0.721**	
BI	0.447^**^	0.531^**^	0.384^**^	0.484^**^	**0.732**

The bold values in the diagonal row are the square roots of the average variance extracted for the constructs in the research model. ***p < 0.001, **p < 0.01, *p < 0.05.

Given the adequate sample size in this study (*n* = 306), a chi-square to degrees of freedom ratio (χ^2^/df) below 5 was considered acceptable, following the criteria recommended by Hu et al. The results indicate that the measurement model demonstrates a satisfactory fit, with *χ*^2^/df = 1.246, AGFI = 0.926, TLI = 0.983, CFI = 0.986, and RMSEA = 0.028 (see Table 4).

**Table 4:** The goodness of fit indices for the measurement model and research model.

Model	*χ* ^2^	*χ*^2^/df	AGFI	TLI	CFI	RMSEA
Measurement model	155.78	1.246	0.926	0.983	0.986	0.028
Research model	196.603	1.524	0.908	0.964	0.970	0.041
Recommended criteria	*p* > 0.05	<5.0	>0.9	>0.9	>0.9	<0.8

### 4.2 The structural model

**Figure 4:**
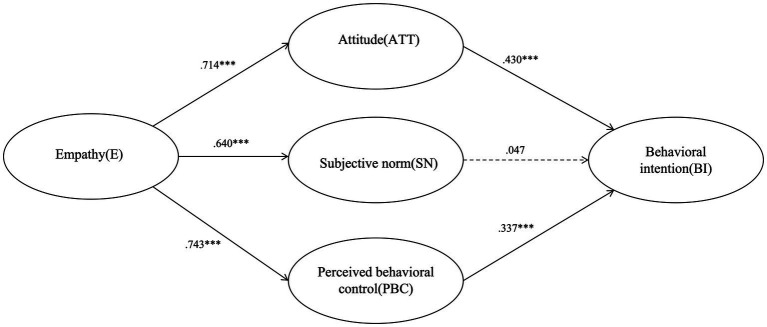
The model and standardized path coefficients. Dashed lines indicated no significant effects. ^***^p < 0.001, ^**^p < 0.01, ^*^p < 0.05.

A structural equation modeling approach was adopted to test the proposed theoretical model. The model exhibited an acceptable fit, with *χ*^2^/df = 1.524, AGFI = 0.908, TLI = 0.964, CFI = 0.970, and RMSEA = 0.041 (see Table 4). The hypothesis testing results indicate that, except for H5, all other hypotheses were supported (see Table 5). Empathy was revealed to impose a significant positive effect on attitude (*β* = 0.714, *p* < 0.001), subjective norm (*β* = 0.640, *p* < 0.001), and perceived behavioral control (*β* = 0.743, *p* < 0.001), supporting H1, H2, and H3. Attitude was significantly and positively associated with behavioral intention (*β* = 0.430, *p* < 0.001), and perceived behavioral control also showed a positive effect on behavioral intention (*β* = 0.337, *p* < 0.001), supporting H4 and H6. In contrast, the impact of subjective norm on behavioral intention was not statistically significant, leading to the rejection of H5. The validated structural model is presented in Figure 4.

**Table 5:** The result of hypothesis test.

hypotheses	Hypothesized path	*B*	*β*	S.E.	*t*	Result
H1	E → ATT	0.744	0.714	0.089	8.379^***^	Supported
H2	E → SN	0.742	0.640	0.093	7.975^***^	Supported
H3	E → PBC	0.779	0.743	0.095	8.208^***^	Supported
H4	ATT → BI	0.457	0.430	0.086	5.342^***^	Supported
H5	SN → BI	0.045	0.047	0.066	0.677	Rejected
H6	PBC → BI	0.356	0.337	0.087	4.077^***^	Supported

^***^p < 0.001, ^**^p < 0.01,^*^p < 0.05. E, empathy; ATT, attitude; SN, social norm; PBC, perceived behavior control; BI, behavior intention.

### 4.3 Indirect effect assessment

This study employed the Bootstrap method to test the indirect effect of empathy (E) on behavioral intention (BI), using a 95% confidence interval that does not include 0 as the criterion for significant effect.

The final results showed that the indirect effect of empathy on behavioral intention through attitude (ATT) was significant (*β* = 0.34, *p* < 0.001, 95% CI = [0.192, 0.563]); the indirect effect of empathy on behavioral intention through subjective norms (SN) was not significant (*β* = 0.033, *p* = 0.588, 95% CI = [−0.091, 0.154]); the indirect effect of empathy on behavioral intention through perceived behavioral control (PBC) was significant (*β* = 0.277, *p* < 0.001, 95% CI = [0.112, 0.491]). In summary, the mediating effects of attitude and perceived behavioral control between empathy and behavioral intention were supported, while the mediating effect of subjective norms was not supported (Table 6).

**Table 6:** Significance testing of indirect effects with bootstrap.

Indirect effect	*β*	S.E.	*p*-value	95% Confidence interval		Significance (*p* < 0.05)
E → ATT → BI	0.34	0.092	0	0.192	0.563	Yes
E → SN → BI	0.033	0.062	0.588	−0.091	0.154	No
E → PBC → BI	0.277	0.096	0	0.112	0.491	Yes

E, empathy; ATT, attitude; SN, social norm; PBC, perceived behavior control; BI, behavior intention.

## 5 Discussion

The findings suggest that the public shows a strong intention to protect endangered animals, with 81% of respondents expressing a willingness to engage in such behavior. (i.e., the mean score of the four items measuring behavioral intention was 4.05 on a 5-point scale).

The results showed that empathy had a strong positive effect on attitude, subjective norm, and perceived behavioral control. In addition, attitude and perceived behavioral control exerted moderate positive effects on behavioral intention.

However, this study is based on individuals’ reported intentions for environmental actions, rather than actual environmental behaviors. Although in the “Theory of Planned Behavior,” intention is regarded as the most direct predictor of behavior, the gap between intention and behavior widely confirmed in environmental psychology indicates that intentions do not always translate into actual behavioral changes. In study by Kollmuss and Agyeman (2002), which clearly identified the well-documented intention-behavior gap in environmental psychology. Even when individuals hold strong pro-environmental intentions, they often fail to translate such intentions into actual behavior due to internal barriers (e.g., habits, perceived control, emotional factors) and external barriers (e.g., costs, time, infrastructure, social norms).

Therefore, these research results should be interpreted as evidence of changes in behavioral intentions, rather than confirmed instances of animal protection behavior. Future studies should incorporate behavioral measurement or longitudinal designs to further validate the observed effects.

### 5.1 Significant association between empathy and attitude, perceived behavioral control, subjective norm

The research results indicate that empathy has a significant positive correlation with attitudes towards the protection of endangered animals, perceived behavioral control, and subjective norms.

Firstly, there is a positive correlation between empathy and the concept of protecting endangered animals. The standardized regression coefficient reaches 0.714. This might be because empathy enables people to emotionally connect with the suffering of endangered species, thereby enhancing their emotional evaluation of animal protection. When people feel concern and sympathy, they are more likely to form a positive attitude towards actions aimed at alleviating such suffering.

Secondly, empathy is also related to subjective norms. The standardized regression coefficient reaches 0.64. People with a higher level of empathy tend to be more sensitive to others’ viewpoints and expectations. This enhanced social awareness may make them more aware of the social pressures and normative expectations related to animal protection, which is also reflected in stronger subjective norms. However, despite the significant relationship between empathy and subjective norms, the mediating role of subjective norms in linking empathy to behavioral intention was not supported. This suggests that although empathetic individuals are more sensitive to social expectations, such perceived social pressure does not necessarily translate into stronger behavioral intentions.

It is worth noting that empathy has a positive impact on perceived behavioral control, and among all the factors, its influence is the most significant. The standardized regression coefficient is as high as 0.743. One possible explanation for this is that empathy care may prompt individuals to develop a stronger sense of personal responsibility and autonomy. The reason is that when individuals perceive a certain issue as closely related to themselves, they may feel more capable of taking action, thereby enhancing their sense of control over their own behaviors.

These findings suggest that empathy, as a potential psychological driving force, plays a crucial role in strengthening individuals’ cognitive evaluation and perceived control of their own behaviors. In other words, the higher the level of empathy, the stronger the perceived behavioral control of the individual, thereby indirectly promoting behavioral intentions.

The conclusion is that decision-makers and designers should introduce a design approach centered on empathy when formulating intervention plans to promote public participation in the protection of endangered animals, because this can effectively enhance the animal protection behavioral intentions of the public.

### 5.2 The impact of subjective norm is not significant

The research results indicate that subjective norms do not have a significant effect on behavioral intentions. This finding is consistent with prior studies.

Previous studies have shown that subjective norms have the least significant impact on behavioral intention (Yan, 2014). Consistent with this, the present study proved that social pressures from family, friends, and broader social groups do not significantly affect the public’s willingness to protect endangered animals.

One possible explanation is that behavior aimed at protecting wildlife is often driven by individual values and empathy rather than external social pressure. In other words, few people take action to protect animals solely because of social pressure; such decisions ultimately depend on the public themselves. Another explanation is that the questionnaire-based interactive experience employed in this study is highly personalized and lacks a discussion component, allowing participants to engage privately without being influenced by the opinions of others. Consequently, the absence of this social context may have further diminished the influence of subjective norms. Furthermore, as this study included empathy as a key variable, the focus of the analysis may have shifted towards individual emotional and attitudinal factors, thereby reducing the relative influence of subjective norms within the model.

### 5.3 Attitude, perceived behavioral control positively predicts behavior intention

The result indicated that both attitude towards endangered animals and perceived behavior control are positively associated with behavior intention to protect animals. The results is in line with prior studies on reducing animal consumption (Zhang et al., 2022). This indicates that favorable attitudes and a higher sense of ease in performing the behavior increase individuals’ likelihood of engaging in wildlife protection. Therefore, the public’s confidence in engaging in animal welfare activities depends primarily on their individual knowledge, access to such activities, and financial resources. If the government can ensure these conditions are met, this will directly enhance the public’s willingness to participate in animal welfare initiatives.

### 5.4 Implications for practice

These findings offer new insights into the conservation of endangered species. The analysis suggests that a variety of factors influence the public’s willingness to protect wildlife. Based on this, the following implications are proposed:

Firstly, given that empathy has a significant influence on the public’s attitudes towards endangered animals, and that attitudes can in turn significantly predict behavioral intentions, future policymakers should prioritize the development of empathy-based communication strategies in their efforts to raise public awareness of wildlife conservation, rather than relying on superficial slogans. For example, designers could integrate empathy into their design practices. When audiences encounter digital works featuring narratives or scenarios closely linked to their own experiences, they are more likely to become immersed in the content. Within the context of new media, designers could apply empathy theory to digital design formats such as personality-based games, virtual reality (VR) and augmented reality (AR) gaming experiences, thereby more effectively eliciting users’ empathetic responses and deepening their understanding of animal conservation themes. Governments could also invest in VR and AR exhibitions, such as simulating the natural habitats of endangered animals—including forests, deserts and wetlands—within 3D environments. Such immersive scenarios would allow the public to experience wildlife habitats first-hand through interactive storytelling (including personality tests and situational interactions), thereby facilitating ‘close-up’ encounters with endangered species. In educational settings, teachers should move beyond the limitations of textbook-based teaching and adopt interactive narrative methods. For example, students could take on the roles of endangered animals and discuss in groups the difficulties these animals might face in the wild. Such experiential narratives help students gain a deeper understanding of the animals’ living conditions. By enhancing empathetic responses, these interventions can effectively foster a positive public attitude towards wildlife conservation.

Secondly, empathy can also enhance an individual’s sense of control over their own behavior; in other words, the more compassionate a person is, the more confident they are in their ability to contribute to animal conservation, which in turn indirectly promotes the intention to act in the protection of endangered animals.

This suggests that future interventions by authorities should not only evoke emotional concern but also emphasise the feasibility of taking action. Currently, in many instances, the public lacks the channels, information and resources required to engage in conservation activities; this is one of the reasons why many people are reluctant to participate in wildlife conservation. Consequently, governments and society should provide clear guidance, actionable steps and accessible channels for participation. Specific measures include wildlife knowledge quizzes, free educational lectures on animal conservation, financial support for conservation activities (such as subsidies), and the establishment of various conservation projects. These measures can boost individuals’ confidence in their ability to contribute, thereby influencing their behavioral intentions.

Thirdly, although public empathy towards endangered animals has a significant impact on subjective norms, subjective norms do not effectively predict behavioral intentions regarding animal conservation. This suggests that relying solely on social pressure or normative information (such as ‘I believe my friends or family would encourage me to care about endangered animals’) may not effectively promote behavioral change among the public. This suggests that future strategies should focus more on intrinsic motivation rather than external expectations. As the public is not influenced simply because those around them are participating in animal conservation actions, future policymakers and programmed designers should pay greater attention to the individual intentions of each member of the public; changing individuals’ inner thoughts is of greater importance.

## 6 Conclusion

This study contributes to the extant research in several respects. Firstly, it enriches the existing literature by identifying the indirect relationship between empathy and wildlife conservation within the framework of the Theory of Planned Behavior (TPB), and by demonstrating the applicability of personality-based tests in the context of endangered species conservation. The results indicate that empathy, as an external variable, has a significant influence on attitudes, subjective norms and perceived behavioral control. In turn, attitudes and perceived behavioral control significantly and moderately predict behavioral intention, while subjective norms do not have a notable effect on behavioral intention.

These findings suggest that the current lack of public engagement in wildlife conservation may be partly due to an insufficient emphasis on empathy-related and internal drivers. This provides policymakers and practitioners with more targeted insights, helping to clarify which aspects should be prioritised in future endangered species conservation strategies.

Secondly, this study provides a reference model for future research. The finding that subjective norms are not significantly related to behavioral intentions suggests that alternative factors, particularly internal variables such as attitudes and perceived behavioral control, should be further explored when studying the public’s intentions regarding wildlife conservation.

Given the crucial role the public plays in the conservation of endangered animals, effectively engaging and motivating individuals is of paramount importance. From a practical perspective, governments and relevant organizations should be encouraged to develop more wildlife conservation programmes, such as interactive, game-based experiences that immerse the public in simulated animal habitats. Such an approach may enhance empathy and deepen understanding of endangered species, ultimately fostering more proactive conservation intentions.

## 7 Research limitation and future work

There are several principal limitations to this research. First, this research only focuses on participant’s behavioral intention on the TPB model but did not measure their long-term animal protection behavioral in real life, this may lead to a discrepancy between intention and actual behavior, commonly referred to as the intention–behavior gap, which has been widely discussed in environmental psychology literature. As a result, the findings should be interpreted with caution and claims regarding the promotion of actual conservation behavior should be limited. While behavioral intention is an important predictor, it does not necessarily translate into sustained real-world actions.

Future research should incorporate longitudinal designs or behavioral tracking methods (e.g., follow-up surveys, in-game behavioral logs, or real-world intervention studies) to better examine whether and how intentions induced by gamified experiences can lead to actual pro-environmental behaviors over time.

Second, the research was carried out in a sole self-test game scenario for animal protection. The impact of empathy arousal and behavioral intention may be limited to this specific game design and cannot be fully generalized to other types of educational games or animal welfare themes.

Third, the sample of this study was mainly comprised of young users and students, who may exhibit relatively higher levels of empathy and awareness of wildlife conservation. In addition, the overall sample size was relatively limited, which may affect the generalizability and robustness of the findings. Future research is therefore encouraged to involve a more diverse and larger sample to improve the representativeness of the results.

Finally, in this study, participants viewed a gameplay video rather than interacting with the game directly, which may limit the ecological validity. In future research, it is recommended that a fully interactive game be developed, allowing participants to actively play and experience the game rather than only watching videos. Collecting data after participants have genuinely engaged with the game would further improve the accuracy and reliability of the results.

## Data Availability

The raw data supporting the conclusions of this article will be made available by the authors, without undue reservation.

## Footnotes

1
https://www.wjx.cn/
