# Supplementary material for: Research on gamified personality test design for wildlife conservation: integrating the theory of planned behavior and empathy
Source: Front Psychol. 2026 Jul 2;17:1839884. doi: 10.3389/fpsyg.2026.1839884 (PMC13372322; doi:10.3389/fpsyg.2026.1839884)
Supplement: Supplementary file 1 [file Table_1.DOCX]

Appendix A.

Table S1. Description of Animal Stimuli

| Animal | Protection Level | Distribution | Category | Characteristics | Reasons for Endangerment |
| --- | --- | --- | --- | --- | --- |
| Armadillo | Vulnerable | The Americas (South America, Central America, the southern part of the United States) | Mammal | Wearing bone armor, it can curl up into a ball when encountering danger. | Habitat destruction, illegal hunting, traffic-related deaths, diseases(Desbiez and Attias, 2022) |
| Saiga Antelope | Critically Endangered | Central Asian grasslands (Kazakhstan, Mongolia, Xinjiang, China) | Mammal | The nose is swollen like an elephant's trunk, and the males have curved long horns. | Poaching (for horns), habitat fragmentation, diseases(Hanski et al., 2023) |
| Malayan Tapir | Endangered | Southeast Asia (Malay Peninsula, Sumatra, Thailand, Cambodia) | Mammal | The body is black and white in color, with a long nose resembling that of a baby elephant. | Deforestation, habitat loss, illegal hunting(Lim et al., 2025) |
| California Condor | Critically Endangered | The west coast of the United States (California, Arizona) | Bird | The entire body is black, and the head is bare without feathers. | Ingestion of lead ammunition by prey, low reproduction rate, habitat reduction(Walters et al., 2010) |
| Blobfish | Vulnerable | The deep sea areas of Australia and Tasmania | Aquatic | The body is gelatinous, and when it emerges from the water, its face droops like a "crying face". | moves extremely slowly and cannot avoid the trawl net, and is often pulled up along with it.(Petrescu-Mag, 2023) |
| Shoebill/Balaeniceps rex | Vulnerable | Central Africa's tropical wetlands (Sudan, Congo, Uganda) | Bird | The mouth is huge and thick, resembling a wooden boot. | Wetlands drying up, habitat destruction, human interference(Mirembe, n.d.) |
| Quokka | Vulnerable | The islands in the southwest of Australia | Mammal | Small in size, with a "smile" on its face | Habitat reduction, predation by invasive species, human interference(Tores et al., 2007) |
| Salamander | Endangered | Mountain streams in East Asia and China | Amphibian | Smooth and moist on the body surface, resembling a lizard but without scales | Water pollution, habitat destruction, illegal capture(Luedtke et al., 2023) |

Table S2. measurement instrument

| variable | description | source |
| --- | --- | --- |
| Empathy (E) | I can understand the helpless feeling of endangered animals. (E1) |  |
|  | When I see endangered animals being harmed, I feel sad.(E2) | (Washio et al., 2019) |
|  | I have sympathy for the endangered animals that have been harmed. (E3) |  |
| Attitude(ATT) | I think participating in wildlife protection actions is beneficial.(ATT1) |  |
|  | I think participating in protection actions is valuable for society.(ATT2) | (Zhang et al., 2022) |
|  | Participating in protection actions makes me feel positive.(ATT3) |  |
|  | Participating in wildlife protection actions is something worth investing time in.(ATT4) |  |
| Social norm(SN) | The animal protection atmosphere in society will prompt me to carry out animal protection actions.(SN1) | (Zhang et al., 2022) |
|  | The current policies' expectations will make me want to protect endangered animals even more.(SN2) |  |
|  | I think my friends or family will encourage me to pay attention to those animals on the verge of extinction.(SN3) | (Zhang et al., 2022) |
|  | The group I belong to advocates that everyone should love wild animals.(SN4) |  |
| Perceived behavior control(PBC) | I can decide for myself whether to protect endangered animals or not.(PBC1) | (Zhang et al., 2022) |
|  | I think I have the conditions to protect endangered animals.(PBC2) |  |
|  | If I want to, I will protect endangered animals.(PBC3) |  |
| Behavior intention(BI) | In the future, I will be willing to participate in activities related to wildlife protection.(BI1) |  |
|  | I will continue to follow the policies related to protecting animals.(BI2) | (Zhang et al., 2022) |
|  | I will try to participate in activities to protect wild animals.(BI3) |  |
|  | In the future, I will strive to participate in activities related to the protection of endangered animals.(BI4) |  |
